# An integrated assessment of water-energy-food benefits from small hydropower refurbishment in rural China

**DOI:** 10.1016/j.isci.2026.116133

**Published:** 2026-05-28

**Authors:** Jiawen Li, Guiliang Tian, Zheng Wu, Xinyu Liu, Qing Xia, Canran Lu

**Affiliations:** 1 Business School, Hohai University, Nanjing, China; 2 Division of Geography and Tourism, Department of Earth and Environmental Science, KU Leuven, Leuven, Belgium; 3 School of Economics and Finance, Hohai University, Changzhou, China; 4 Yangtze River Protection and Green Development Research Institute, Nanjing, China

**Keywords:** Energy resources, Urban planning, Water resource engineering

## Abstract

Small hydropower, deeply embedded in rural China, plays an important role in poverty alleviation, energy transition, and rural development. However, the integrated benefits of refurbishment remain insufficiently understood. To address this issue, this study integrates the qualitative Gioia method, a game-theory-based weighted DEMATEL model, and a multi-attribute decision-making method based on Gray Pythagorean Fuzzy Hesitant Fuzzy Sets to construct a water-energy-food integrated benefit evaluation framework. The results show that small hydropower refurbishment forms a water-energy-food integrated benefit structure characterized by water as the nexus, energy as the medium, and food as the anchor. Benefits show clear functional differentiation, with management capacity improvement, ecological flow regulation, and irrigation coordination as driver benefits; social reputation and agricultural production stability as feedback benefits; while women’s participation and greenhouse gas reduction represent peripheral benefits. Evaluation cases from five regionally representative plants show that geographical context, refurbishment design, and community infrastructure affect refurbishment benefits.

## Highlights

• An integrated framework was developed to identify and assess SHP benefits
• SHP refurbishment formed an interlinked water, energy, and food benefit structure
• Management of ecological flow and irrigation coordination were key drivers
• SHP benefits varied markedly across plants under different local conditions

## Graphical abstract

**Figure:**
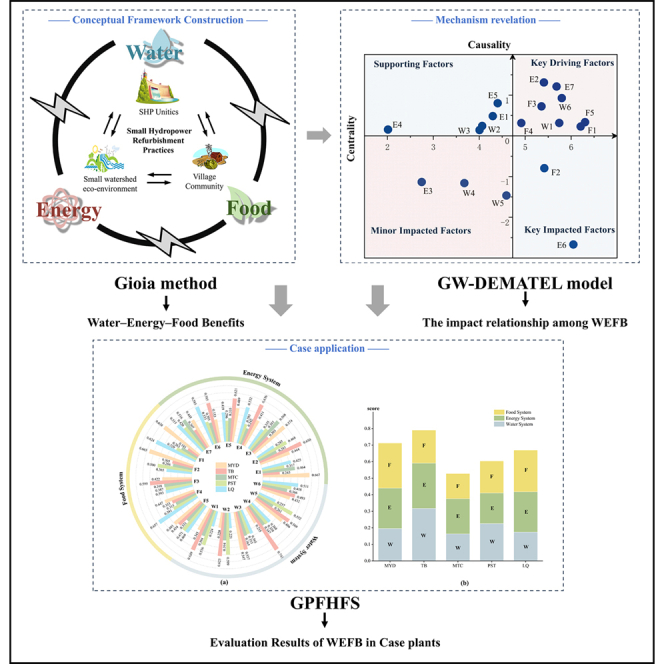

## Teaser

Energy resources; Urban planning; Water resources engineering

## Introduction

Globally, hydropower has long been regarded as the most widely developed and highest-efficiency form of clean renewable energy, forming a crucial component in addressing the climate crisis and ensuring the future of a low-carbon energy supply.^1^ Among its various forms, small hydropower (SHP)-with advantages such as low investment requirements, strong adaptability, and flexible decentralized deployment—can effectively reach remote regions with low-density energy supply. SHP not only reduces greenhouse gas emissions but also offers opportunities to revitalize local communities.^2^ generating direct structural linkages with multiple Sustainable Development Goals (SDGs), including affordable and clean energy (SDG7), clean water and sanitation (SDG6), and the elimination of rural poverty and hunger (SDG1 and SDG2).^3^ China is a global leader in SHP development, defining SHP as hydropower plants below 50,000 kW. SHP has played a significant role in rural poverty alleviation, job creation, and livelihood improvement, producing notable regional development outcomes.^4^ However, the widespread and intensive construction of SHP has altered natural river flows, impaired riverine ecosystem health, intensified water-use competition, and, combined with aging hydropower equipment, introduced considerable safety concerns.^5^ In 2016, China’s No. 1 Central Document explicitly called for the “development of green small hydropower,” prompting the transformation of some SHP into green small hydropower (GSHP) demonstration plant projects through targeted refurbishment.^6^ As current policy directions move away from isolated demonstration assessments toward comprehensive enhancement, a key scientific question emerges: How do small hydropower refurbishment practices (SHRP) reshape functional attributes and thereby create an interlinked water-energy-food benefit (WEFB) structure? Furthermore, how the heterogeneity, interdependencies, and shared generative mechanisms of these benefits manifest across different plants will critically influence the feasibility and scaling pathways of GSHP development. Accordingly, it is necessary to establish an integrated analytical framework capable of systematically identifying the multidimensional benefits of SHRP, mapping the structure of WEFB, and uncovering their underlying generative mechanisms. Such a framework will not only test the feasibility of SHRP in advancing GSHP development but also provide strategic guidance for further comprehensive improvement of demonstration plants.

Existing studies typically examine the externalities of engineering projects and classify the comprehensive benefits of SHP into five dimensions: economic, social, ecological-environmental, managerial, and technological. The economic dimension primarily focuses on the financial feasibility of SHP projects^7^ and the implementation of green finance instruments.^8^ The social dimension addresses resident satisfaction,^9^ social equity outcomes,^10^ and the improvements in community welfare.^11^ The ecological-environmental dimension addresses impacts on aquatic organisms,^12^ land use,^13^ and ecosystem services,^14^ while the managerial dimension pertains to plant-level production organization, operational coordination,^15^ and community consultation mechanisms.^16^ The technological dimension focuses on system optimization and the development of intelligent operation^17^ and maintenance systems.^18^ Although such externality-based frameworks facilitate the evaluation of multidimensional impacts, they tend to produce parallel and static representations of benefits. The functional attributes of SHP-storing runoff, regulating water resources, supporting agricultural irrigation, and simultaneously improving rural energy accessibility indicate that its benefits do not exist in isolation. Rather, SHP generates interdependent and interacting linkages across water regulation, energy supply, and agricultural production, forming a systemic structure within the water-energy-food nexus (WEFN). Regarding the construction of benefit frameworks, existing research largely relies on literature-based synthesis or expert-driven logic to define benefit components. For example, Li et al. (2025) employ a procedural grounded theory approach to identify the benefit structure of SHP refurbishment.^19^ While the method offers strong operational advantages, data interpretation remains unavoidably shaped by researchers’ prior cognitive frames, thereby limiting the potential to detect mechanisms or relationships. Building on inductive benefit frameworks, methodological choices vary according to research purposes and data availability. Multi-criteria decision analysis (MCDA) is commonly applied to multi-stakeholder evaluation problems and incorporates fuzziness to quantify impacts or benefits that are difficult to measure, thereby supporting decision-making.^20^ Cost-benefit analysis combined with financial indicators is used to assess economic feasibility and return on investment.^21^ Life cycle assessment (LCA) evaluates the full life cycle impacts of SHP construction and refurbishment.^22^ Energy analysis assesses energy flows involved in project construction or upgrading and can convert these flows into monetary equivalents.^23^ Survey-based methods are also widely used for the empirical analysis of social perceptions related to SHP projects.^24^ Despite their respective strengths, these approaches generally rely on researcher-defined indicator systems and emphasize “evaluation” rather than “discovery,” making it difficult to uncover the deeper mechanisms underlying refurbishment activities. Moreover, in multi-stakeholder settings, traditional MCDA depends on deterministic scoring and struggles to capture cognitive divergence, hesitation, and fuzziness among residents, managers, and experts, limiting the accurate representation of cross-stakeholder perceptions.

**Figure 1:**
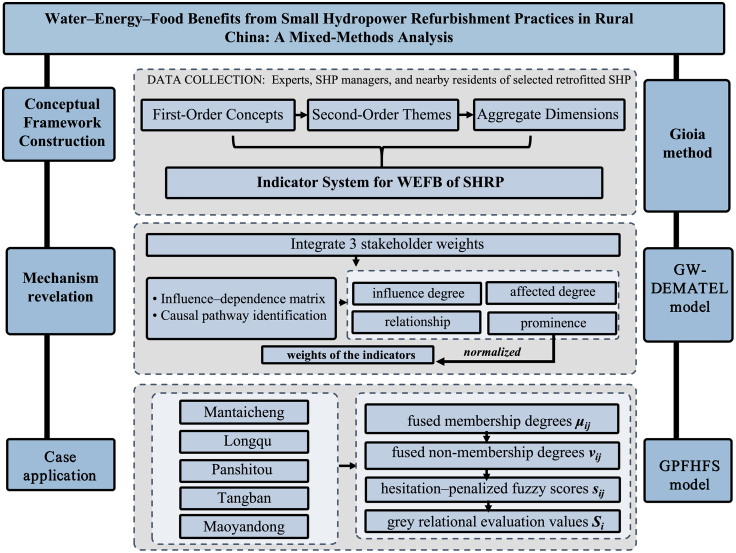
Research framework for evaluating water-energy-food benefits of small hydropower refurbishment

Re-examining the benefits of SHRP from the perspective of functional attributes and systemic linkages is not only theoretically meaningful but also practically valuable for guiding integrated resource management. However, notable content and methodological gaps remain in the existing literature. Compared with conventional analytical approaches, the Gioia method enables researchers to extract emerging concepts and construct theoretical structures directly from interview materials through a systematic coding procedure, thereby minimizing the influence of preconceived logic on analytical outcomes.^25^ This method is particularly suitable for research contexts in which theoretical foundations are underdeveloped or key mechanisms have not yet been fully revealed. Therefore, introducing a theory-building pathway grounded in the Gioia methodology is expected to provide a more open, rigorous, and discovery-oriented analytical basis for understanding the systemic benefits of SHRP. In parallel, the multi-attribute decision-making approach based on Gray Pythagorean Fuzzy Hesitant information is capable of integrating cognitive divergences among different evaluation stakeholders and mitigating subjective uncertainty.^26^ This approach demonstrates strong scientific robustness when dealing with hesitation, inconsistency, and ambiguity in subjective judgments during the assessment of SHP functional benefits. Accordingly, this study integrates the strengths of these methodological tools and establishes the research framework shown in Figure 1. First, interviews and material collection are conducted with experts, SHP plant managers, and surrounding residents involved in the selected SHRP cases. The spatial distribution of the 35 case plants is presented in Figure S1. Through the Gioia method, we develop an emergent benefit-explanation framework and construct a more open, insightful theoretical model of SHPRP benefits. Second, based on the theoretical framework, an evaluation indicator system is developed, and a game-theory-based weighted DEMATEL model is used to synthesize the views of the three stakeholder groups and identify the weights and interrelationships among benefit factors. Finally, the Gray Pythagorean Fuzzy Hesitant Fuzzy Sets (GPFHFS) is applied to evaluate the benefits of representative SHP plants. The basic characteristics of the interview sample are summarized in Table S1.

The novelty of this study is reflected in three dimensions: theory, methodology, and practice. First, at the theoretical level, the study draws on the Gioia method to extract concepts and relationships from first-hand interview data, uncovering a unique WEFB structure inherent in SHP refurbishment. It further clarifies the multidimensional coupling mechanisms linking water-resource regulation, clean energy provision, and agricultural production support. These findings provide direct micro-scale evidence for Hoff’s (2011) WEF Nexus framework and expand its applicability to regional resource-system research, offering a theoretical lens for understanding the role of small-scale hydropower infrastructure within multi-resource systems.^27^ Second, at the methodological level, the study develops an interdisciplinary analytical framework integrating Gioia-based theory generation, GW-DEMATEL causal-structure identification, and GPFHFS evaluation. This framework overcomes the limitations of existing studies that rely on expert-prescribed indicators, struggle to reveal benefit-generation mechanisms, and inadequately handle cognitive divergence and fuzzy hesitation among stakeholders. The proposed approach enables a complete analytical process-from mechanism discovery and causal-chain construction to multi-stakeholder comprehensive evaluation-and offers a generalizable toolset for assessing benefits in complex socio-ecological-technical systems. Finally, at the practical level, the study synthesizes heterogeneous perspectives from experts, SHP plant managers, and local residents. Through game-theoretic combined weighting and hesitant fuzzy information processing, it develops a sensitive and discriminative comprehensive evaluation instrument. This tool supports performance diagnosis, governance optimization, and the selection of follow-up refurbishment pathways for SHP projects. Moreover, it identifies key benefit factors and bottlenecks in refurbishment activities and provides methodological support for the policy design and scaling of green SHP demonstration plants.

## Results

### Conceptual framework construction for WEFB of SHRP

Based on the contextual characteristics of SHPR, various associated benefits were systematically identified and categorized. First, a first-order term analysis was conducted by identifying terms, codes, and categories from the raw data, including reports on SHRP and interview materials from the three stakeholder groups, followed by the merging of similar categories. Second, a second-order theme analysis was performed based on the initial concepts, extracting theoretical-level constructs and summarizing them into 14 core benefit themes, which encompass improvements in resource system functionality and the expansion of social co-benefits. Based on both levels of analysis, second-order themes were further aggregated and organized to construct a data structure diagram that progressively reveals the theoretical interpretation, as shown in Table 1. Through the three-stage coding process, the resource attributes and functional roles of SHRP were clarified. It was found that SHP, through its interactions among plants, villagers, and ecosystems, generates water resource benefits, energy system benefits, and food system benefits by linking water, energy, and food functions.

**Table 1:** Gioia coding results of WEFB in SHRP

First-Order Concepts (partial)	Second-Order Themes	Aggregate Dimensions
“The river has never been this stable in years; now there’s water all year round.”/“Ecological flow release guarantees downstream ecosystems even in dry seasons.”/“We get a subsidy of 0.02 RMB per ton of water.”	Water supply	Water system benefits
“Integrated water purification equipment was added; pipelines replaced with PE pipes; biochemical pools built to protect water quality.”	Water quality improvement	
“The landscape was restored; eroded land treated; vegetation regrown on hillsides.”/“The renovation helped control landslides.”	Soil and water conservation capacity enhancement	
“People used to say there were no fish. Now some come here to fish.”/“Over a million fish fry are released annually in coordination with fisheries authorities.”	Biodiversity enhancement	
“The plant looks like a park now. On weekends people come to fish and take photos.”/“Aesthetic weirs and leisure spaces have been built; locals use them regularly.”	Landscape and ecosystem services	
“Real-time monitoring equipment was installed to ensure ecological flow discharge.”	Ecological flow regulation	
“Now each cubic meter of water generates more power. Just running the machine makes money.”/“The renovated plant is centrally dispatched to fill supply gaps.”	Power generation efficiency improvement	Energy system benefits
“We can monitor the plant on our phones now. No need to go on-site when issues occur.”	Intelligent operation and maintenance	
“Hydropower replaces coal and significantly reduces greenhouse gas emissions.”/“The increased use of clean energy greatly contributes to carbon reduction.”	Greenhouse gas emission reduction	
“My wife now works as a control operator at the plant. She says even tech jobs can be hers.”/“Local women were employed at the plant with equal pay and balanced gender ratios.”	Female participation in technical work	
“My son studied hydropower and now works at my company.”/“Training and ethics education are emphasized to build professional capacity.”	Human capital development	
“Now the villagers treat the plant as a park. Some even come here to film TikTok videos.”/“It helps increase farmers’ income and enrich rural life.”	Social reputation and recognition	
“We participated in exchange visits to improve management and clarify responsibilities.”/“We’ve standardized safety protocols and refined operations.”	Management capacity enhancement	
“Whenever needed, the plant opens the gate to release water for free use by villagers.”	Agricultural irrigation guarantee	Food system benefits
“We can now plant a second rice crop, thanks to stable water access. The plant prioritizes our use.”	Agricultural timing and productivity stability	
“The downstream irrigation canals were reinforced and sealed for better water delivery.”	Agricultural infrastructure improvement	
“Discounted electricity and free water from the reservoir have long been provided to nearby residents.”	Public utilities synergy improvement	
“Water allocation is adjusted upstream and downstream to avoid conflicts.”	Coordinated irrigation mechanisms	

#### Water system benefits

The water system benefits of SHPR derive from comprehensive reservoir rehabilitation and the intelligent upgrading of related equipment, and are manifested across six dimensions: water supply, water quality improvement, soil and water conservation capacity enhancement, biodiversity enhancement, landscape and ecosystem services, and ecological flow regulation. Water supply benefits originate from in-depth channel rehabilitation along both sides of river sections and extensive desilting operations in reservoirs. These measures reduce water wastage, increase usable inflows, and improve water supply efficiency. Several SHP plants significantly enhanced their water intake performance through intake expansion, dredging, and gate refurbishment—for example, the TP plant increased its design diversion flow by 83.5%, while the ZJL plant improved its intake capacity to 12.96 m^3^/s. During interviews, feedback such as “noticeable reduction in water abandonment” appeared 184 times, demonstrating strong consistency between engineering records and perceived improvements. Water quality improvement results from river channel remediation, installation of trash racks, debris removal devices, and online water quality monitoring systems, complemented by the establishment of river patrol teams responsible for routine inspection and waste clearance. Such upgrades were added at many plants (including GF, TP, CH), and more than 20 out of 35 plants established regular river-patrol mechanisms. Interview comments such as “the water is clearer” appeared frequently, providing direct qualitative confirmation of the engineering data. Soil and water conservation capacity is enhanced through vegetation restoration around river channels and reservoir areas, slope stabilization, and ecological rehabilitation. These measures strengthen flood control, increase surface roughness, and improve soil infiltration. Many plants—including CH, MJ, and GF—implemented large-scale afforestation and bank reinforcement, reducing landslide risks and sedimentation. Corresponding interview feedback, such as “the riverbank is more stable” and “less sediment,” appeared approximately 152 times, aligning closely with the documented scope of rehabilitation works. Biodiversity enhancement stems from fish restocking programs and habitat restoration measures. Annual fish stocking at many plants (e.g., MYD and PX) involved the release of hundreds of thousands to over one million juvenile fish. Moreover, without compromising flood safety, plants excavated fast-flowing river sections to restore water depth during dry periods, increase water surface area, and create diverse habitats such as deep pools and shallow shoals. Corresponding local feedback—such as “more fish” and “people come to fish”—appeared 157 times, validating the ecological improvements. Landscape and ecosystem services are enhanced through afforestation, vegetation landscaping, and water surface purification measures, which refreshed the appearance of plant areas and improved water clarity. plants such as GF, TP, and CH carried out plant-area greening, water-surface cleaning, and riverside landscape improvements. Interview comments such as “like a park” and “many people come on weekends” appeared more than 120 times, reflecting the tangible aesthetic and recreational benefits generated by refurbishment. Ecological flow regulation is achieved by installing automated ecological flow monitoring and control systems, ensuring compliance with ecological discharge standards and enabling eligibility for ecological tariff subsidies. Several plants—including TP, GF, and YDH—installed automated ecological flow monitoring systems, while many others added ecological generating units that allow simultaneous ecological flow release and power generation. Interview feedback such as “there is still water during the dry season” and “ecological flow is stable” appeared over 98 times, fully consistent with the documented technological upgrades.

#### Energy system benefits

The energy system benefits of SHPR arise from equipment upgrades that enhance the technological capabilities of SHP plants, thereby promoting localized energy accumulation and human capital development. These benefits can be categorized into seven dimensions: power generation efficiency improvement, intelligent operation and maintenance, greenhouse gas emission reduction, female participation in technical work, human capital development, social reputation and recognition, and management capacity enhancement. Power generation efficiency improvement is primarily driven by upgrades to turbines, generators, and electrical equipment, along with the installation of microcomputer monitoring systems and industrial video systems that improve operational efficiency. Typical plants such as MJ, PX, and JSTT reported overall efficiency increases of 6%–15%, with MJ achieving levels above 83.7%. Interview responses such as “more stable generation” and “greater output” appeared 198 times, showing strong alignment between perceptual evidence and engineering data. Intelligent operation and maintenance are realized through the installation of digital dashboards, integration into regional centralized control centers and maintenance platforms, and the deployment of smart systems to support automated production and safety management. Numerous plants—including TB, MBZ, and DP—installed computer-based monitoring systems, industrial video systems, and digital “smart dashboards.” More than 25 out of 35 plants achieved the “unattended or minimally attended operation” standard. Related interview expressions appeared 122 times, confirming the widespread adoption of smart O&M practices. Greenhouse gas emission reduction results from the more efficient utilization of hydropower as a renewable energy source, thereby decreasing reliance on fossil fuels and reducing emissions of carbon, sulfur, and other greenhouse gases. The refurbishment increased hydropower utilization efficiency, reduced water abandonment, and raised the share of renewable electricity. Some plants generated an additional 2–5 million kWh of clean electricity per year, directly contributing to CO_2_ reduction. Interview statements such as “more environmentally friendly” and “less coal consumption” appeared 199 times, reinforcing the environmental significance of SHPR. Female participation in technical work reflects SHPR’s contribution to gender equity in the energy sector. Many plants—including TB and MYD—created new technical positions for women and offered targeted training programs. Interview responses such as “women can do technical work” and “more job opportunities” appeared 122 times, consistent with the labor-structure transformation following automation and digitalization upgrades. Human capital development is reflected in improvements to the physical working environment, enhancement of employee welfare, and the provision of ongoing technical training. Facility upgrades and equipment renewal improved comfort and safety in the workplace, and many plants—including PX, GF, and JSTT—conducted systematic training programs. Interview feedback such as “better technical skills” and “more training opportunities” appeared 174 times, demonstrating the accumulation of human capital in remote SHP plants. Social reputation and recognition are enhanced through inter-plant outreach, exchanges, and publicity activities that elevate the visibility of SHP plants and improve public perceptions. After refurbishment, many plants attracted media coverage, resident visits, and cross-plant learning exchanges. Interview remarks such as “more widely recognized” and “others come to learn from us” appeared 85 times, reflecting the strengthened social legitimacy of green SHP development. Management capacity enhancement is demonstrated through the adoption of standardized safety procedures during refurbishment, revision of internal management systems, and implementation of safety training and awareness campaigns. Many plants—including GF, TB, and CH—completed safety-standardization reforms and upgraded operational procedures and management workflows. Interview feedback such as “more standardized” and “clearer management” appeared 173 times, aligning with the documented institutional reforms.

#### Food system benefits

The food system benefits of SHPR arise from its role in strengthening the localized integration of water and energy infrastructure with agricultural systems in mountainous regions. Building on the water-to-energy conversion capacity of SHP, SHPR provides critical support for agricultural production and rural livelihoods. These benefits can be categorized into five dimensions: agricultural irrigation guarantee, agricultural timing and productivity stability, agricultural infrastructure improvement, public utilities synergy improvement, and coordinated irrigation mechanisms. Agricultural irrigation guarantee is reflected in the rehabilitation and upgrading of irrigation water delivery networks by many SHP plants, reducing water losses during irrigation and expanding irrigation coverage. These improvements buffer agricultural production from seasonal precipitation fluctuations. Several plants—including PST, TB, and GF—restored or newly constructed irrigation channels, effectively expanding irrigated areas and reducing water loss. Interview statements such as “water is still available during droughts” appeared 116 times, providing strong qualitative validation for the engineering interventions. Agricultural timing and productivity stability are enhanced through upgraded pump-irrigation systems enabled by SHPR, which provide stable, clean energy for agricultural operations. Improved reliability of electricity supply reduces energy-related disruptions and contributes to agricultural mechanization. plants such as MJ and PX now provide more stable power for key agricultural periods, reducing vulnerability to outages. Corresponding feedback—such as “no fear of power outages during peak farming seasons”—appeared 57 times, confirming the benefits perceived by farmers. Agricultural infrastructure improvement is demonstrated through the construction and maintenance of irrigation canals, reinforcement of rural roads and farmland areas, and the procurement or donation of agricultural machinery. plants such as TB and PX constructed canals, strengthened rural roads, and donated farming equipment. Interview responses—such as “roads are easier to travel” and “the canals have been repaired”—appeared 182 times, aligning with the documented refurbishment measures. Public utilities synergy improvement reflects SHPR’s contribution to integrated water-energy support for agriculture and related public infrastructure. Several plants—including MYD, TP, and GF—covered village electricity bills, supplied water free of charge, or improved communal infrastructure. Interview accounts such as “water use is free” and “lower electricity costs” appeared 110 times, illustrating how SHPR strengthens the provision of public utilities for rural communities. Coordinated irrigation mechanisms are embodied in strengthened communication channels between SHP plants and farmers, ensuring that crop irrigation needs receive priority. Many plants—including PST and TP—established irrigation-priority arrangements and facilitated upstream-downstream negotiation over agricultural water allocation to safeguard food production. Interview responses—such as “our irrigation is guaranteed first” and “no more quarrels over water”—appeared 98 times, confirming the effective functioning of these coordination mechanisms.

### Key element analysis of WEFB in SHRP

**Figure 2:**
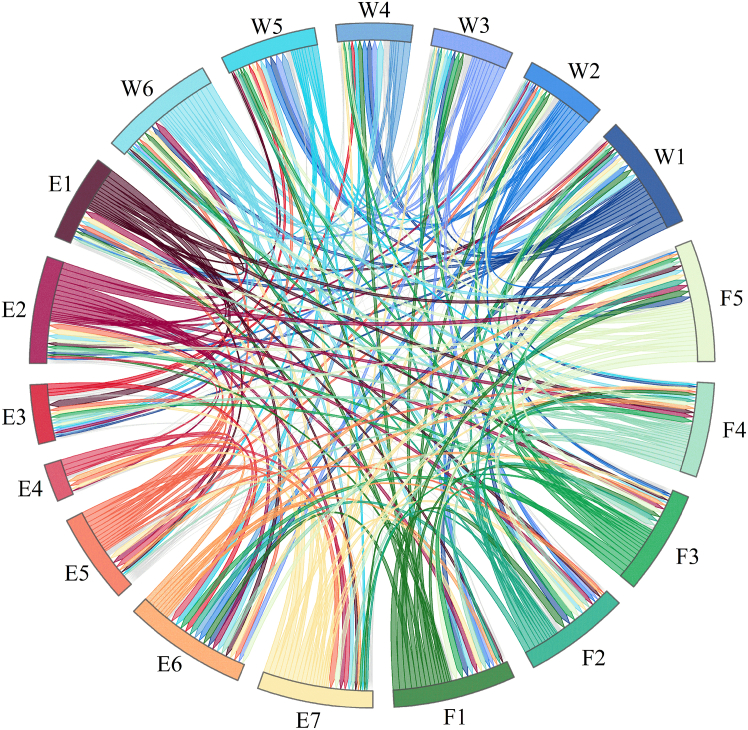
Impact relationships among water-energy-food benefits in small hydropower refurbishment

To further analyze the interrelations among the WEFB of SHRP, the constituent dimensions and meanings of the water, energy, and food systems are first identified. Evaluation indicators for each WEFB dimension are designed based on the aggregate dimensions and second-order themes identified in the coding process. The second-order themes represent the specific components included in each benefit dimension. Subsequently, based on Equations 1, 2, 3, 4, 5, 6, 7, 8, 9, 10, static mapping indices and corresponding weights for each indicator are calculated, as shown in Table 2. A chord diagram is drawn based on the fused original matrices of experts, plant managers, and local residents to visualize the mutual influence relationships among the benefits, as illustrated in Figure 2.

**Table 2:** Indicator system for WEFB of SHRP

Overall benefit dimension	Indicators	No.	D	C	P	R	Weight
Water system benefits	Water supply	W1	3.0296	2.7115	5.7411	0.3181	0.0662
	Water quality improvement	W2	2.1536	1.9101	4.0637	0.2435	0.0469
	Soil and water conservation capacity enhancement	W3	2.0745	1.9307	4.0052	0.1438	0.0462
	Biodiversity enhancement	W4	1.2585	2.4172	3.6758	−1.1587	0.0424
	Landscape and ecosystem services	W5	1.5639	3.0267	4.5907	−1.4628	0.0530
	Ecological flow regulation	W6	3.3621	2.4336	5.7957	0.9286	0.0669
Energy system benefits	Power generation efficiency improvement	E1	2.3900	1.9036	4.2937	0.4864	0.0495
	Intelligent operation and maintenance	E2	3.3600	2.0473	5.4074	1.3127	0.0624
	Greenhouse gas emission reduction	E3	0.8072	1.9393	2.7465	−1.1321	0.0317
	Female participation in technical work	E4	1.0841	0.9283	2.0124	0.1558	0.0232
	Human capital development	E5	2.6018	1.7991	4.4009	0.8027	0.0508
	Social reputation and recognition	E6	1.6904	4.3576	6.0479	−2.6672	0.0698
	Management capacity enhancement	E7	3.4487	2.2350	5.6837	1.2137	0.0656
Food system benefits	Agricultural irrigation guarantee	F1	3.2187	2.9894	6.2081	0.2294	0.0716
	Agricultural timing and productivity stability	F2	2.3122	3.1045	5.4167	−0.7923	0.0625
	Agricultural infrastructure improvement	F3	3.0395	2.3150	5.3545	0.7246	0.0618
	Public utilities synergy improvement	F4	2.6155	2.2997	4.9152	0.3158	0.0567
	Coordinated irrigation mechanisms	F5	3.3203	2.9822	6.3024	0.3381	0.0727

The influence degree and affected degree reflect the comprehensive effect of benefit factors, which are classified into three tiers using Jenks natural breaks based on Table 2, and analyzed in conjunction with the relationships depicted in Figure 2. In terms of influence degree, the high-value group (3.0296–3.4487) includes E7, W6, E2, F5, F1, F3, and W1 in descending order, indicating that these seven factors exert the greatest influence on other benefits in the WEFB of SHRP. The medium-value group (2.0745–3.0296), including F4, E5, E1, F2, and W2, suggests that these five factors have a relatively strong impact on other WEFB. The low-value group (0.8072–2.0745) includes W3, E6, W5, W4, E4, and E3, suggesting these six factors have relatively limited influence on other benefit factors. Regarding the affected degree, the high-value group (2.9894–4.3576) consists of E6, F2, and W5, indicating these three factors are most affected by other benefit factors. The medium-value group (2.0473–2.9894) includes F1, F5, W1, W6, W4, F3, F4, and E7, suggesting they are considerably affected by other factors. The low-value group (0.9283–2.0473) comprises E2, E3, W3, W2, E1, E5, and E4, indicating these factors are relatively less affected by others.

**Figure 3:**
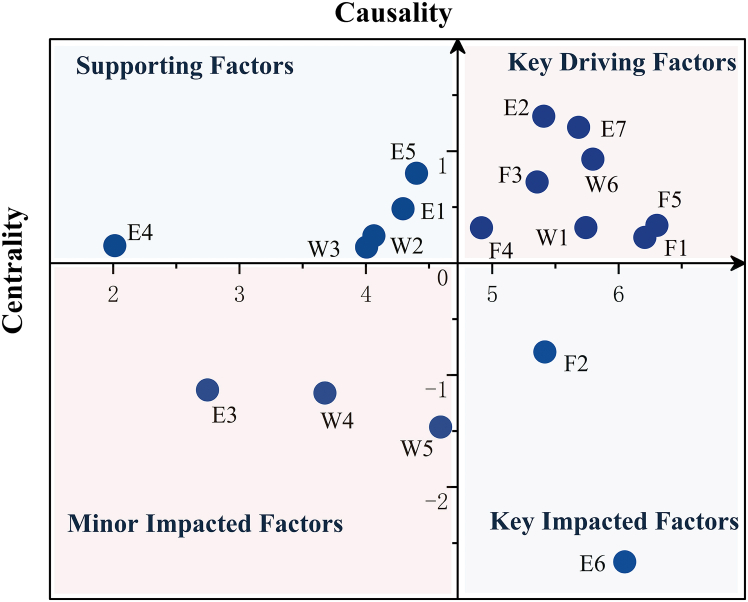
Relationship and prominence quadrant diagram of benefit factors

The relationship and prominence, respectively, reflect the causal tendency of benefit factors and their overall interaction intensity within the system. Using the average centrality (4.8145) and average cause degree (0) as vertical and horizontal axis thresholds, the factors are distributed across four quadrants, as shown in Figure 3. This quadrant chart is used to classify key factors and reveal the core driving forces and trade-offs within the WEFB. F5, F1, W6, W1, E7, E2, F3, and F4 fall in the first quadrant, exhibiting high centrality and positive cause degree. These factors significantly influence WEFB in SHRP, act as primary causal variables, and are the key driving forces. E5, E1, W2, W3, and E4 lie in the second quadrant with low centrality but positive cause degree. They can influence other factors but have a limited overall impact, typically serving a supporting role. E3, W4, and W5 are positioned in the third quadrant with low centrality and a negative cause degree. These factors are affected by others, but the degree of influence remains limited. F2 and E6 belong to the fourth quadrant, characterized by high centrality and negative cause degree. These two factors are highly susceptible to influence from other benefit factors.

In summary, SHRP does not merely lead to localized technological optimization or isolated economic gains; rather, they reconfigure institutional foundations and reshapes resource pathways, thereby establishing a synergistic and symbiotic benefit network that spans ecological environmental quality, clean energy provision, and agricultural security. Internally, improvements in management capacity, ecological flow regulation, and the coordination of irrigation water use function as core benefit drivers. These foundational mechanisms facilitate the joint advancement of multiple subsystem outcomes—such as agricultural irrigation security, water supply assurance, and enhanced power generation efficiency—highlighting the central role of water-energy resource allocation, mediated through management and institutional mechanisms, in driving system-wide benefit improvement. Meanwhile, benefits such as social reputation and the stability of seasonal agricultural capacity primarily operate as external performance indicators of SHP operation, reflecting public perception and the feedback signals associated with agricultural production stability. These elements serve as important social metrics for evaluating the perceived effectiveness and legitimacy of the refurbishment process. Although some peripheral ecological and social benefits currently exhibit limited influence within the system, their latent importance should not be underestimated. Particularly in the context of future sustainability and equity agendas, these factors hold the potential to evolve into key system-level objectives.

### Evaluation of WEFB in the case plants of SHRP

**Figure 4:**
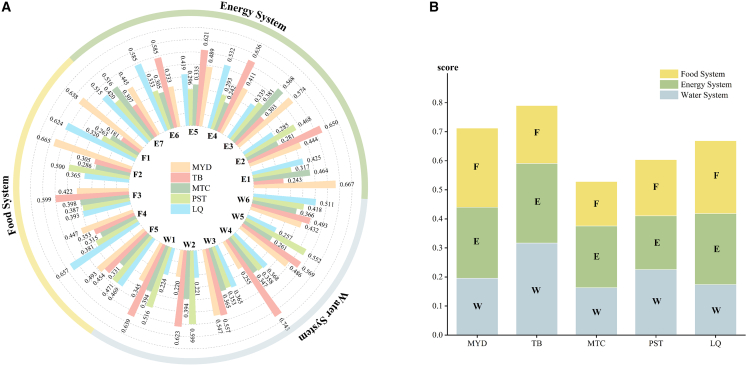
Evaluation results of water-energy-food benefits in the case plants (A) Scores of individual benefit indicators for the case plants. (B) Scores of subsystem benefits and overall integrated benefits for the case plants.

Based on the score consistency method, the evaluations from experts, plant managers, and local residents were integrated. Using Equations 12, 13, 14, 15, 16, 17, 18, 19, 20, 21, and 22, the comprehensive evaluation results of the five case plants were obtained. Table 3 further presents each plant’s average membership degree μ, non-membership degree ν, hesitancy degree π, the corresponding gray relational coefficients γᵢ, and the final comprehensive scores Sᵢ. The hesitancy degrees for TB, MYD, LQ, PST, and MTC are 0.033, 0.032, 0.035, 0.032, and 0.030, respectively, indicating that the overall evaluation model is stable and the multi-stakeholder fusion is robust, thereby providing reliable decision-support value. The results show that TB, MYD, and LQ exhibit significantly higher comprehensive scores than PST and MTC, suggesting that their post-refurbishment performance in the multidimensional water-energy-food benefits is notably stronger. The detailed comparison is illustrated in Figure 4.

**Table 3:** Comprehensive evaluation results of the five SHP plants based on the fused multi-stakeholder scores

Parameter	*μ*	*ν*	*π*	*γ*_*i*_	*S*_*i*_
MYD	0.8042	0.5784	0.0320	0.7045	0.7124
TB	0.8118	0.5682	0.0328	0.7971	0.7903
MTC	0.7531	0.6509	0.0298	0.5305	0.5285
PST	0.7822	0.6165	0.0318	0.5920	0.6043
LQ	0.7833	0.6048	0.0348	0.6437	0.6696

TB achieved the highest overall WEFB score of 0.7903, demonstrating the strongest performance among all plants and forming a virtuous “water–energy” feedback loop through its SHRP. Its water system benefits score (0.3170) was supported by consistently strong performance across all indicators, reflecting the plant’s emphasis on ecological water management and restoration—likely attributable to its core role as a regional water supply provider. The energy system score (0.2741) was also substantial, driven by notable achievements in female participation, human capital development, and social reputation, indicating concerted efforts to promote gender equity and capacity-building. Although TB invested in agricultural infrastructure, its food system benefits remained relatively limited, given the low dependence on agriculture in the coastal region where the plant is located.

MYD ranked second with a score of 0.7124, characterized by a synergistic “energy–food” interaction. Its highest-performing dimension was the food system benefits (0.2726), reflecting improvements in irrigation infrastructure and water-use efficiency in mountainous farming contexts. MYD also performed strongly in energy system benefits, particularly in power generation efficiency, emissions reduction, and human capital development, consistent with its generation-oriented functional profile. Its water system strengths lie in soil conservation, ecosystem services, and ecological flow regulation.

LQ ranked third with a score of 0.6696, displaying a renovation pattern centered on enhancing irrigation security. Its food system benefits (0.2515) reflected improvements in irrigation coverage, public facility co-development, and coordinated water-use mechanisms. Energy system benefits were driven by gender participation, reputation enhancement, and management capacity strengthening—aligned with the socio-institutional conditions of northwest China, where water scarcity heightens the importance of governance. LQ’s strongest water system indicator was ecological flow regulation, contributing to watershed stability.

PST ranked fourth with a score of 0.6043, without a distinctly dominant subsystem. Its water system benefits were moderate, largely attributable to reservoir capacity improvements and ecological facility upgrades. PST exhibited strong performance in intelligent O&M but showed low power generation efficiency due to scale constraints and dispatch limitations. The plant supported agricultural timing and irrigation reliability through local coordination but lacked a solid foundation in agricultural infrastructure.

MTC had the lowest overall score (0.5285), indicating partial improvements but insufficient systemic synergy. Its water system benefits (0.1633) were low across all indicators. The energy system score (0.2123) reflected strengths in generation efficiency and emissions reduction—largely stemming from automation upgrades—but was accompanied by weak institutional and social dimensions. The food system score (0.1528) highlighted limited integration with agricultural activities and weak water-energy-agriculture linkages.

## Discussion

This study integrates qualitative and quantitative approaches by employing the Gioia method, together with the WEFN theory, to construct a theoretical framework for the WEFB of SHRP. A GW-DEMATEL model with game-theoretic combination weighting was then applied to analyze the micro-level interaction mechanisms among these benefits. Finally, the gray Pythagorean hesitant fuzzy multi-attribute decision-making method was used to evaluate the WEFB of five representative SHP plants. Through this combined approach, the study aims to elucidate the benefits of SHRP from the perspective of resource interlinkages and provide analytical support for the comprehensive enhancement of SHP.

## An interpretable and quantifiable evaluation framework for the WEFB

After clarifying the interaction mechanisms underlying the WEFB generated by SHRP, a key challenge lies in how to structure and quantify these mechanisms—particularly in micro-scale settings characterized by limited data availability, complex behavioral dynamics, and implicit feedback loops—and ultimately develop a comparable evaluation system. Existing WEF evaluation approaches primarily rely on macro-level statistics, process-based simulation, LCA, or single-actor engineering acceptance systems. While effective under conditions of robust data availability, these approaches are difficult to apply directly to SHP plants in remote mountainous areas, where data are sparse, actors are diverse, and institutional dependence is strong.^28^ In contrast, the methodological framework adopted in this study demonstrates unique adaptability to micro-scale contexts and constitutes an evaluation paradigm with broader applicability. First, the Gioia coding procedure provides a bottom-up pathway from mechanism identification to indicator construction, ensuring that the evaluation structure emerges from the “practical logic of actors” rather than from indicators transplanted directly from literature. This differs from many previous WEF studies that define indicators largely through theoretical assumptions.^29^ The mechanism structure identified here arises organically from actual operations, institutional arrangements, and community interactions, thus exhibiting greater explanatory power for differences among individual plants and better capturing the embedded relationships between smallholder irrigation practices and SHP dispatch behavior. Second, the game-theoretic combination weighting DEMATEL model offers an endogenous mechanism for generating multi-actor weights, enhancing the interpretability of system interactions. Mainstream engineering evaluation or cost-benefit analysis frameworks often overlook resident behavior and local institutions,^20^ while single-actor expert-based methods may overestimate the knowledge of particular actor groups.^24^ By incorporating a game-theoretic equilibrium among three types of actors—experts, plant managers, and residents—this study enables irrigation needs, ecological perceptions, and everyday water-use behaviors of rural households to become structural components of the system. This enriches the relational analysis of WEFB and addresses the commonly reported omission of social dimensions in prior WEF research. Third, GPFHFS combined with gray relational analysis enables cross-plant comparability under small-sample conditions, overcoming one of the most prominent limitations in micro-scale evaluation. Methods such as LCA, emergy analysis, or energy-water scenario modeling require high-frequency operational data and detailed engineering parameters, which are typically unavailable for remote SHP plants. The hesitant fuzzy structure used in this study captures cognitive uncertainty—reflecting the ambiguity that actors genuinely face when full information is lacking—while gray relational analysis constructs a comparative space across different benefit types, allowing TB, MYD, PST, MTC, and LQ to be evaluated under a unified structural framework. Fourth, compared with existing case-based studies, the “structural difference-pathway difference” uncovered here provides theoretical insights. Many evaluations of SHP merely report descriptive findings such as “ecological improvement,” “enhanced power generation,” or “higher resident satisfaction.” In contrast, this study demonstrates—through causal-structural analysis—that high-performing plants form a positive “water-energy-food” interaction loop, whereas low-performing plants fail to close this loop. This shifts the analytical paradigm from listing engineering outcomes to identifying systemic mechanisms, enabling differences in benefits to be interpreted as structural consequences rather than accidental outcomes.^30^^,^^31^ Consequently, the findings offer a pathway-oriented framework to guide future engineering design, institutional arrangements, and community governance, and provide a structured reference for cross-regional or cross-type comparisons in subsequent research.

### Mechanism identification of the WEFB generated by SHRP

Based on the three-stage coding procedures of the Gioia method, this study identifies a system-level WEFB structure generated by SHRP—one that positions water as the foundational linkage, energy as the enabling medium, and food as the stabilizing pivot. Specifically, water system benefits emphasize ecological restoration and water environmental security; energy system benefits reflect technological empowerment and organizational capacity enhancement; and food system benefits concentrate on safeguarding agricultural production and strengthening institutional coordination mechanisms. Together, improvements within each subsystem generate a synergistic uplift effect. While this mechanism aligns with the general logic of the WEFN highlighted in existing studies,^32^ it exhibits distinct operational dynamics and practice-based triggers. The unique contribution of this research lies not only in identifying the benefits of SHRP at the hydropower-plant level but also in revealing the systemic interactions formed by SHP as a micro-scale resource node embedded in remote rural contexts. First, water resources act as the precondition and controlling factor for the entire system, serving as the fundamental origin of both energy and food benefits within SHRP. Interview evidence and case materials show that increases in diversion flow, ecological flow regulation, and reservoir desilting establish the hydrological foundation for electricity generation—illustrating a typical “water-to-energy” resource provisioning relationship. While Afshar et al. (2022) conceptualize the water system as a supply-side constraint on energy and food at macro scales,^33^ this study uncovers how such constraints are operationalized at the micro-infrastructure level—through reservoir dispatching rules, real-time monitoring systems, and daily operational protocols rather than through policy-level arrangements. Similarly, water availability directly supports agricultural irrigation and seasonal production scheduling, forming a “water-to-food” relationship that is more fine-grained and more dependent on localized institutional arrangements than macro-level models can capture. Second, the energy system influences the food system primarily through “stability” rather than “quantity” at the small scale. Unlike macro-level literature, which focuses on energy supply-demand equilibrium or carbon-emission scenarios,^34^ the energy benefits of micro SHP manifest as improved power supply stability, reduced operational disruptions due to automation, and enhanced reliability of energy use in agricultural production. Case evidence shows that a stable electricity supply after SHRP supports pump irrigation, agricultural machinery, and small-scale processing activities—making the energy subsystem a critical regulator of agricultural resilience. This “energy-to-food” enabling relationship is structurally significant yet triggered through mechanisms fully distinct from macro-scale models, relying instead on turbine efficiency, intelligent O&M, and localized energy autonomy. Third, feedback responses from the food system constitute the most distinctive reverse mechanism within the WEFB framework. Irrigation demands, cropping calendars, water-use disputes, and perceptions of ecological flow collectively shape operational decisions at SHP plants, forming a “food-to-water/energy” behavioral feedback loop. Such mechanisms are largely absent from existing Nexus literature, as macro-scale models cannot capture highly contextualized, day-to-day behavioral adjustments.^32^ This study provides micro-scale evidence that the agricultural system is not merely a passive recipient of water and energy but actively reconfigures reservoir dispatch logic through usage behavior and local norms—thereby forming the “interrelations and interactions” loop emphasized by Afshar et al. (2022).^33^ In addition, whereas existing studies often define “food” in terms of crop yield, trade, or cropping patterns,^34^ at the SHP scale the food subsystem is more appropriately conceptualized as the stability of agricultural production conditions, including irrigation reliability, seasonal scheduling, infrastructure status, and cultivation environments. This definition better reflects the resource realities of smallholder farming in mountainous regions and enhances the explanatory power of the WEFB concept. It is also noteworthy that this study incorporates the ecological benefits associated with fish restocking into the water system dimension, thereby strengthening the conceptualization of the “water-food” linkage.^35^ Thus, this study moves beyond descriptive observation to construct a mechanism framework that is theoretically consistent with existing Nexus scholarship while retaining strong practical explanatory capacity within micro hydropower infrastructure contexts. The framework demonstrates that SHRP functions as a “resource reconfiguration node” in which engineering, institutional, and behavioral elements are jointly coupled to achieve coordinated interactions among the water, energy, and food subsystems. This finding addresses a notable gap in the WEF literature regarding micro-scale mechanisms and provides an actionable pathway for applying Nexus theory in infrastructure governance practice.

### Relationships among the WEFB of SHRP and analysis of evaluation results

The structured analysis reveals that the benefits generated by SHRP do not accumulate in a linear or additive manner; rather, they emerge through a directional and coherent system pathway. Within this pathway, key benefit components do not exert influence uniformly but operate along a “driver-transmission-feedback” sequence, forming a recognizable system hierarchy. Improvements in management capacity, ecological flow regulation, and coordinated irrigation water use occupy central positions within this relational structure. These benefits alter resource allocation patterns and reshape the interactions between SHP plants, ecosystems, and farming households. In contrast, factors such as social reputation or seasonal agricultural capacity stability, which are highly influenced but weakly causal, function more as feedback outcomes of system operation. Although these factors do not directly regulate subsystem behavior, they accurately reflect public acceptance and the operational state of agricultural production. Some environmental and social dimensions-such as female participation or greenhouse gas reduction-may appear peripheral under current policy priorities. However, their importance should not be evaluated solely by their degree of influence; rather, greater attention should be placed on these benefits when constructing comprehensive evaluation frameworks. Identifying this hierarchical structure provides critical insights for analyzing the differentiated refurbishment outcomes across plants. The evaluation of five representative SHP plants using the gray Pythagorean hesitant fuzzy multi-attribute decision-making approach further illuminates these structural differences. Due to their location in distinct environmental and socio-institutional settings—spanning northeast, southeast, northwest, southwest, and central China—these plants exhibit heterogeneous performance outcomes. Among the two high-performing plants, TB and MYD, positive WEFB linkages were established through coordinated optimization of water-power-dam functions, aligned with each plant’s primary operational focus. TB, as a water-supply-oriented plant, enhanced its support for power generation and agricultural production through ecological restoration; MYD, primarily generation-oriented, integrated improvements in energy efficiency and emissions reduction with upgrades to irrigation infrastructure. These cases illustrate that when refurbishment pathways capitalize on plant-specific comparative advantages and are supported by institutional, technical, and managerial interventions, high levels of WEFB are more easily achieved. In contrast, PST and MTC remain largely confined to technical upgrades without deep integration into ecological or social systems. Their limited system coupling results in weak benefit realization and inadequate pathway closure. These findings underscore that SHRP must extend beyond conventional hydropower refurbishment toward multi-objective coordination across water, energy, and food systems—tailored to the plant’s primary function and regional geographic constraints—to support sustainable SHP operations.^36^ Collectively, these insights advance our understanding of how SHRP generates WEFB and extend the generalizability of the findings. On one hand, the structured pathway developed in this study provides a unified analytical framework for plants across diverse regions, shifting evaluation from an “engineering input-oriented” perspective to a “system coordination-oriented” perspective. On the other hand, the framework offers micro-level mechanism hypotheses for future WEF Nexus research, including how to design institutional nodes under resource constraints, how to diagnose the conditions required for synergy pathway formation, and how to leverage behavioral feedback to construct adaptive dispatch mechanisms. These emerging propositions are directly grounded in empirical results and provide a foundational basis for future theoretical and methodological development.

### Limitations of the study

Despite its contributions, this study contains several limitations and opportunities for further exploration. First, although China currently has 1,067 green SHP demonstration plants, data availability constraints limited this study to 35 plants for exploratory analysis. Given the diversity and complexity of SHP conditions in remote mountainous regions, future research could expand the sample size to enrich the understanding of WEFB across different contexts. Second, the WEFB identified in this study are derived from analyzing benefit outcomes rather than from modeling the full refurbishment process. In reality, SHRP is inherently processual, involving dynamic interactions over time. Thus, future research should further investigate process mechanisms and develop a more comprehensive representation of the WEF linkages throughout the entire refurbishment cycle. Third, the lack of long-term operational data from SHP plants restricts the application of more data-intensive methods such as energy analysis, LCA, or high-resolution energy-water simulation. The MDCA-based approach used in this study inevitably involves some degree of subjectivity. The evaluation data in SHRP exhibit structural characteristics of “extremely small samples, differentiated actors, linguistic fuzziness, and high hesitancy,” with only 7 experts, 2 plant managers, and 4 villagers participating. Each indicator ultimately yields a hesitant Pythagorean fuzzy triplet, which fundamentally limits the applicability of advanced quantitative methods such as deep learning, structural equation modeling, or Bayesian networks. Future research could apply these more fine-grained analytical methods by integrating multi-source data—such as remote sensing, high-frequency operational data, mobile-based behavioral monitoring, and institutional archives—to enhance the robustness and stability of the evaluation results.

## Resource availability

### Lead contact

Requests for further information and resources should be directed to and will be fulfilled by the lead contact, Guiliang Tian (tianguiliang@hhu.edu.cn).

### Materials availability

This study did not generate new materials.

### Data and code availability

• All data generated or analyzed during this study are included in the manuscript and supplementary tables and figures.
• This paper does not report original code.
• Any additional information required to reanalyze the data reported in this paper is available from the lead contact upon request.

## Acknowledgments

The authors gratefully acknowledge the financial support provided by the 10.13039/501100004543China Scholarship Council for Jiawen Li’s joint training at KU Leuven. We thank the Tangban, Maoyandong, Longqu, Panshitou, and Mantaicheng hydropower plants for their support and assistance during the field investigation. We also thank Professor Maarten Loopmans of the Division of Geography and Tourism, KU Leuven, for his valuable comments and support. Finally, we thank the anonymous reviewers for their constructive comments and suggestions.

## Author contributions

Conceptualization, J.L. and G.T.; methodology, J.L. and Z.W.; investigation, J.L. and Z.W.; validation, J.L., G.T., and Z.W.; writing – original draft, J.L., G.T., and Z.W.; writing—review and editing, G.T., Z.W., X.L., Q.X., and C.L.

## Declaration of interests

The authors declare no competing interests.

## STAR★Methods

### Key resources table

**Table:** 

REAGENT or RESOURCE	SOURCE	IDENTIFIER
**Deposited data**		

Basic information on the 35 refurbished SHP plants	Field investigation and administrative records compiled by the authors	Figure S1 and Table S1
Survey and interview data collected from experts, SHP plant managers, and local residents	Field surveys and semi-structured interviews conducted by the authors	Not publicly deposited due to participant confidentiality; available from the lead contact upon reasonable request
Refurbishment documents of case SHP plants	Project documents and technical reports provided by the case SHP plants	Not publicly deposited due to confidentiality restrictions; available from the lead contact upon reasonable request

**Software and algorithms**		

Microsoft Excel 2021	Microsoft	https://www.microsoft.com/microsoft-365/excel
MATLAB R2023b	MathWorks, Inc.	https://www.mathworks.com/products/matlab.html
ArcGIS Desktop 10.6.2	Esri	https://www.esri.com/en-us/arcgis/products/arcgis-desktop/resources

### Experimental model and study participant details

This study develops a methodological system composed of the Gioia method, GW-DEMATEL, and GPFHFS to address the key characteristics of SHRP, including multi-stakeholder participation, substantial cognitive divergence, complex benefit structures, and limited sample size. Each of the three methods corresponds to different layers of research needs and forms a progressive logical chain, exhibiting strong methodological alignment and distinct advantages.

#### Data and information sources

The research data were primarily obtained from three sources: refurbishment documents from the case SHP plants, semi-structured interview transcripts with experts, SHP plant managers, and local residents, and questionnaire-based evaluation data.

1 The case plants were selected mainly from the 19 demonstration SHP plants included in the China Small Hydropower Efficiency Improvement and Capacity Expansion Project, supplemented by refurbishment reports from GSHP demonstration plants in northern China. In total, 35 refurbished GSHP demonstration plants—covering a wide range of natural geographic conditions and socioeconomic settings across China—were included. Their spatial distribution is shown in Figure S1.
2 The interview materials were collected through 40–60 min semi-structured interviews with 127 respondents, including experts in water resources and hydropower management, managers of the 35 SHP plants, and residents living in the surrounding areas. Respondent characteristics are presented in Table S1. The process of obtaining the interview data consisted of three steps:first, developing an interview framework for identifying the benefits of SHRP; second, conducting interviews in two rounds by different members of the research team, with each session audio-recorded and subsequently transcribed into text; and third reviewing both the interview process and the resulting transcripts to minimize interviewer bias and avoid cognitive preconditioning. After all the materials were collected, the interview corpus was organized and categorized, with one-quarter of the transcripts retained for theoretical saturation testing after the development of the conceptual structure. To derive the mutual influences among benefit dimensions and their integrated weights, all 127 respondents were further asked—during on-site interviews—to score the influence strength between each pair of key benefits based on their own experience. A 0–4 scale was adopted, and all scores were orgnized by the research team afterward.
3 The scoring data for each case plant were obtained from a second-round questionnaire survey conducted after the construction of the final indicator system. The collection of evaluation data followed the operational characteristics of SHP plants and the structural features of the stakeholder groups. SHP plants are typically located in remote mountainous areas, with a limited number of operation and maintenance personnel and highly differentiated job responsibilities. Therefore, for engineering-side evaluation, four individuals (the chief engineer and two technical management team leaders) were selected for each case plant, as they are the only actors capable of providing comprehensive, systematic, and internally consistent technical judgments within the constraints of the plant’s knowledge structure. On the community side, the villages surrounding the case plants are generally small and remote, and only a limited number of residents possess the ability to independently complete questionnaires and maintain stable experiential knowledge of their interactions with the plant in irrigation, ecology, and daily life. To ensure representativeness and validity, four directly affected residents with stable and substantial cognitive experience were included. The expert group consisted of seven professionals with long-term experience in water resources and hydropower engineering. Because the GPFHFS emphasizes relative weighting structures across stakeholder groups, an excessively large expert sample would distort the balance among these groups; maintaining a moderate sample size ensures fair and proportional representation. It is important to emphasize that the objective of this study is to uncover the mechanisms through which SHRP generates WEFB at the microscale. The methodological paradigm therefore aligns with exploratory multi-stakeholder cognitive analysis. In such studies, the priority is to obtain information from actors with deep, direct, and process-based experience rather than to pursue large-sample statistical inference. International studies in related fields—such as water resources management, rural energy infrastructure, and ecological engineering—commonly rely on approximately 10–20 key informants as core samples. Accordingly, the sample size and composition in this study fully conform to established scientific practice in comparable research.

#### Selection and overview of evaluated case plants

For further analysis, one representative plant was selected from each of the five major regions-Northeast, Northwest, Central, Southeast, and Southwest—for evaluation. These plants exhibit significant regional variation in both natural geography and SHRP schemes, as well as in levels of community engagement. The evaluation of these five plants enables the analysis of WEFB across regions, providing insights for region-specific promotion of SHRP. Specifically, Mantaicheng(MTC) in Wangqing County, Yanbian Korean Autonomous Prefecture, Jilin Province, is a diversion-type hydropower plant. It has a dam length of 337 m and a height of 37 m, with a total reservoir capacity of 99.9 million m^3^ and an installed capacity of 24,900 kW. The plant primarily generates electricity while also supporting urban water supply, river rehabilitation, aquaculture, and tourism. Longqu(LQ), located in Ganzhou District, Zhangye City, Gansu Province, is a non-regulating diversion-type plant that diverts water through low dams or weirs. With an installed capacity of 16,000 kW, it primarily generates electricity, while also ensuring ecological flow, agricultural irrigation, and urban water supply. Panshitou(PST), located in Dahejian Township, Qibin District, Hebi City, Henan Province, is a dam-toe SHP built on Panshitou Reservoir. The dam is 606 m long and 102.2 m high, with a total capacity of 608 million m^3^ and an installed capacity of 9,380 kW. Its primary functions are flood control and water supply, with additional roles in irrigation, power generation, and aquaculture. Tangban(TB), located in Tangban Village, Pandu Township, Lianjiang County, Fujian Province, is a run-of-river plant with a reservoir capacity of 9.53 million m^3^ and an installed capacity of 12,000 kW. It primarily generates electricity and also contributes to water supply for Fuzhou and Lianjiang. Maoyandong(MYD), located in Yongning Township, Luxi County, Honghe Prefecture, Yunnan Province, is a diversion-type plant with a dam height of 14.4 m and a reservoir capacity of less than 1 million m^3^. It has an installed capacity of 15,000 kW and primarily focuses on electricity generation, while also serving functions in irrigation, water regulation, and ecological conservation.

### Method details

#### Identifying benefit-generation mechanisms in SHRP using the Gioia method

The Gioia method enables the systematic identification of benefit-generation mechanisms directly from interview materials without relying on pre-defined indicators. This feature makes it particularly suitable for SHRP, a research context characterized by strong situational dependence and mechanisms that remain insufficiently theorized. Compared with other qualitative approaches, the Gioia method’s three-stage coding process offers greater transparency and stronger theoretical elevation, providing a traceable conceptual foundation for subsequent quantitative modeling. This study applied the procedural approach of the Gioia method to analyze the benefits arising from multi-actor interactions in SHRP, thereby uncovering the WEFB of SHP. The Gioia method employs a structured three-stage coding process that, while faithfully capturing field context, progressively ascends to a theoretical construction level, ultimately producing a highly explanatory and well-structured process model.^37^ Specifically, in the first stage, concepts are extracted directly from the raw data using respondents’ own “first-order terms,” preserving both their semantic content and contextual characteristics. In the second stage, comparisons among first-order concepts are used to identify latent logical relationships, gradually abstracting them into second-order themes—facilitating the transition from “phenomenon language” to “theoretical language.” The third stage aggregates second-order themes into aggregate dimensions that explain the underlying mechanisms of the observed phenomena, thus achieving a leap from concrete observations to theoretical abstraction. The validity and robustness of the coding process were tested through theoretical saturation, which was reached when no new concepts emerged. Moreover, the coding was independently conducted by three researchers, and researcher triangulation was ensured through repeated cross-checking and discussion of discrepancies.

#### Identifying causal relationships among WEFB factors using the GW-DEMATEL model

The GW-DEMATEL model is employed to characterize the causal relationships among benefit dimensions and quantify the interaction mechanisms across WEFB factors. Compared with the traditional DEMATEL approach, which relies on a single expert’s judgment matrix, this study integrates the perspectives of experts, managers, and residents through a game-theoretic composite weighting scheme. This enhancement enables the systematic reconciliation of heterogeneous stakeholder preferences and substantially improves the robustness of conventional causal-structure analysis. Grounded in graph-theoretical principles, the DEMATEL model identifies the logical relationships among system factors by constructing a direct influence matrix and calculating the degree to which each factor influences or is influenced by others.^38^These values are subsequently used to derive the cause degree and centrality, which together determine the causal structure and relative importance of each factor within the system. To incorporate heterogeneous perceptions into the SHRP analysis, a game-theory-based combination weighting method is applied to fuse the influence matrices provided by different stakeholder groups. By minimizing the deviation between the aggregated matrix and each group’s original judgment matrix, this approach ensures that the integrated causal structure reflects a balanced and mutually consistent representation of expert, managerial, and public perspectives.

##### Elicitation and structuring of stakeholder influence judgments

Each stakeholder group rates the influence strength on a scale from 0 to 4—corresponding to no influence, weak influence, moderate influence, strong influence, and very strong influence. After averaging and normalizing the questionnaire ratings for each group, direct influence matrices for the three stakeholder categories are constructed for a system containing n evaluation indicators:

(Equation 1) $$T^{\left(k\right)}=\left[t_{\text{ij}}^{\left(k\right)}\right]\in R^{n\times n},k=1,2,3$$

where k denotes the stakeholder group (1 = experts, 2 = managers, 3 = residents), $t_{\text{ij}}^{\left(k\right)}$ represents the perceived influence of indicator i on indicator j by group k, and T^(k)^ is the corresponding n\times n direct influence matrix.

##### Game-theoretic fusion of stakeholder influence assessments

To enable unified weighting and fusion, each matrix is vectorized column-wise:

(Equation 2) $$v^{\left(k\right)}=\text{vec}\left(T^{\left(k\right)}\right)\in R^{n^{2}\times 1}$$

where v^(k)^ contains all entries $t_{\text{ij}}^{\left(k\right)}$ stacked into a single vector.

The combination weights for the three stakeholder groups are defined as:

(Equation 3) $$\alpha ={\left(\alpha_{1},\alpha_{2},\alpha_{3}\right)}^{T},\alpha_{k}\geq 0,\sum_{k=1}^{3}\alpha_{k}=1$$

and the fused judgment vector is given by:

(Equation 4) $$\omega =\sum_{k=1}^{3}\alpha_{k}v^{\left(k\right)}$$

where α_k_ denotes the weight assigned to group k, α is the weight vector, and ω is the fused indicator-pair vector of dimension n^2^×1.

##### Optimization of stakeholder weights via least-squares minimization

To ensure that the fused judgment approximates the overall preference of the three groups, an average reference vector is introduced:

(Equation 5) $$\bar{\omega }=\frac{1}{3}\sum_{k=1}^{3}v^{\left(k\right)}$$

and a combination matrix is constructed:

(Equation 6) $$W=\left[v^{\left(1\right)},v^{\left(2\right)},v^{\left(3\right)}\right]\in R^{n^{2}\times 3}$$

where $\bar{\omega }$ is the arithmetic mean of the three judgment vectors and W is the matrix composed of these vectors.

The optimal weights are obtained by solving the following least-squares problem, which identifies the weight vector α that minimizes the deviation between the fused judgment and the benchmark preference:

(Equation 7) $$\min_{\alpha }\|W\alpha -\bar{\omega }{\|}_{2}^{2}$$

##### Construction of the integrated DEMATEL matrix and derivation of influence indices

The resulting optimal fused judgment vector is then computed as:

(Equation 8) $$\omega^{*}=W\alpha^{*}$$

and reshaped back into matrix form:

(Equation 9) $$T^{\text{final}}=\text{reshape}\left(\omega^{*},n,n\right)$$

where α^∗^ is the optimal combination weight vector, ω^∗^ is the optimal fused judgment vector, T^final^ is the final DEMATEL total influence matrix of size n×n, and t_ij_ denotes the fused influence of indicator i on indicator j.

Based on T^final^, the influence degree, affected degree, relationship, and prominence are computed as:

(Equation 10) $$\left\{ \begin{array}{c} \begin{array}{c} D_{i}=\sum_{j=1}^{n}t_{\text{ij}}\\ R_{i}=\sum_{j=1}^{n}t_{\text{ji}} \end{array}\\ \begin{array}{c} S_{i}=D_{i}-R_{i}\\ C_{i}=D_{i}+R_{i} \end{array} \end{array}\right.$$

where D_i_ represents the influence degree (outward effect) of indicator i, R_i_ the affected degree (inward effect), S_i_ the cause degree used to determine whether an indicator acts as a cause or an effect, and C_i_the centrality reflecting the overall importance of indicator i within the system.

Finally, the prominence are normalized to obtain the indicator weights:

(Equation 11) $$w_{i}=\frac{C_{i}}{\sum_{j=1}^{n}C_{j}},i=1,2,\cdots ,n$$

where $\sum_{j=1}^{n}C_{j}$ is the total prominence and w_i_ is the normalized importance weight of indicator i, satisfying w_i_≥0 and $\sum_{i=1}^{n}w_{i}=1$. These weights are subsequently used in the GPFHFS-based comprehensive evaluation.

#### Evaluating WEFB performance of SHP plants using GPFHFS

The Pythagorean Hesitant Fuzzy Set, derived from the traditional Pythagorean fuzzy set, accommodates both multi-valued assessments and hesitation inherent in human judgment.^39^ In the evaluation of SHRP, cognitive divergence among experts, managers, and residents as well as incomplete information are more pronounced; hence, PHFS is further enhanced through gray relational analysis to improve robustness under small-sample conditions.^40^ The GPFHFS is well-suited for contexts with limited sample size and evaluation data that exhibit hesitation and incompleteness. It accommodates multi-valued judgments and uncertainty, and its integration with gray relational analysis enhances robustness. Relative to standard fuzzy evaluation models, GPFHFS can more accurately capture fuzzy preference information intrinsic to rural governance settings, thereby providing a more sensitive and stable measurement framework for SHRP performance.

Assume there are m SHP plants and n evaluation indicators, with three stakeholder groups: experts (k = 1), managers (k = 2), and residents (k = 3). The evaluation information provided by group k for plant i under indicator j is expressed as

(Equation 12) $$\omega_{\text{ij}}^{\left(k\right)}=\left(a_{\text{ij}}^{\left(k\right)},b_{\text{ij}}^{\left(k\right)},c_{\text{ij}}^{\left(k\right)}\right),0\leq a\leq b\leq c\leq 1$$

where $a_{\text{ij}}^{\left(k\right)}$, $b_{\text{ij}}^{\left(k\right)}$, and $c_{\text{ij}}^{\left(k\right)}$ denote the lower, middle, and upper bounds of the hesitant membership values given by group k.

##### Consistency-based objective integration

To avoid subjectively imposing equal weights on the three stakeholder groups, a consistency-based integration approach is adopted. The standard deviations of the three components are computed as

(Equation 13) $$\left\{ \begin{array}{c} \sigma_{\text{ij}}^{a}=\text{std}\left(\left\{a_{\text{ij}}^{\left(k\right)}\right\}\right),\\ \sigma_{\text{ij}}^{b}=\text{std}\left(\left\{b_{\text{ij}}^{\left(k\right)}\right\}\right),\\ \sigma_{\text{ij}}^{c}=\text{std}\left(\left\{c_{\text{ij}}^{\left(k\right)}\right\}\right) \end{array}\right.$$

where $\sigma_{\text{ij}}^{a}$, $\sigma_{\text{ij}}^{b}$, and $\sigma_{\text{ij}}^{c}$ represent the dispersion of the three groups’ evaluations for each component.

Consistency-based weights are then assigned as:

(Equation 14) $$\lambda_{\text{ij}}^{\left(k\right)}=\frac{1/\sigma_{\text{ij}}^{b}}{\sum_{k=1}^{3}1/\sigma_{\text{ij}}^{b}+\varepsilon }$$

where ε is a sufficiently small constant introduced to avoid division by zero.

Based on these weights, the fused triplet is obtained as

(Equation 15) $$\left\{ \begin{array}{c} {\bar{a}}_{\text{ij}}=\sum_{k=1}^{3}\lambda_{\text{ij}}^{\left(k\right)}a_{\text{ij}}^{\left(k\right)},\\ {\bar{b}}_{\text{ij}}=\sum_{k=1}^{3}\lambda_{\text{ij}}^{\left(k\right)}b_{\text{ij}}^{\left(k\right)},\\ {\bar{c}}_{\text{ij}}=\sum_{k=1}^{3}\lambda_{\text{ij}}^{\left(k\right)}c_{\text{ij}}^{\left(k\right)} \end{array}\right.$$

where $\left({\bar{a}}_{\text{ij}},{\bar{b}}_{\text{ij}},{\bar{c}}_{\text{ij}}\right)$ constitutes the fused hesitant fuzzy evaluation of plant i under indicator j.

##### Membership and non-membership estimation

The integrated membership degree is calculated using the Simpson area method:

(Equation 16) $$\mu_{\text{ij}}=\frac{{\bar{a}}_{\text{ij}}+4{\bar{b}}_{\text{ij}}+{\bar{c}}_{\text{ij}}}{6}$$

where μ_ij_ denotes the expected membership degree.

The corresponding non-membership interval under the Pythagorean constraint (μ^2^+ν^2^ ≤ 1) is given by:

(Equation 17) $$\left\{ \begin{array}{c} \nu_{\text{ij}}^{U}=\sqrt{\max\left(0,1-{\bar{a}}_{\text{ij}}^{2}\right)},\\ \nu_{\text{ij}}^{M}=\sqrt{\max\left(0,1-{\bar{b}}_{\text{ij}}^{2}\right)},\\ \nu_{\text{ij}}^{L}=\sqrt{\max\left(0,1-{\bar{c}}_{\text{ij}}^{2}\right)} \end{array}\right.$$

where $\nu_{\text{ij}}^{L}$, $\nu_{\text{ij}}^{M}$, and $\nu_{\text{ij}}^{U}$ are the lower, middle, and upper bounds of the non-membership interval.

Similarly, the expected non-membership degree is obtained by

(Equation 18) $$\nu_{\text{ij}}=\frac{\nu_{\text{ij}}^{U}+4\nu_{\text{ij}}^{M}+\nu_{\text{ij}}^{L}}{6}$$

where ν_ij_ represents the aggregated non-membership degree.

##### Hesitation degree and Pythagorean fuzzy score

The hesitation degree is computed as

(Equation 19) $$\pi_{\text{ij}}=\sqrt{\max\left(0,1-\mu_{\text{ij}}^{2}-\nu_{\text{ij}}^{2}\right)}$$

where π_ij_ denotes the residual uncertainty associated with indicator j for plant i.

The final Pythagorean fuzzy score is

(Equation 20) $$s_{\text{ij}}=\mu_{\text{ij}}\cdot \left(1-\nu_{\text{ij}}\right)-\beta \cdot \pi_{\text{ij}}$$

where β is the penalty coefficient for hesitation; in this study, β = 0.3 is adopted to balance score discrimination and stability under multi-source heterogeneity.

##### Gray relational coefficient

The gray relational coefficient is further constructed as

(Equation 21) $$\xi_{\text{ij}}=\frac{\min_{i}\min_{j}|s_{j}^{*}-s_{\text{ij}}|+\rho \;\max_{i}\max_{j}|s_{j}^{*}-s_{\text{ij}}|}{\left|s_{j}^{*}-s_{\text{ij}}|+\rho \;\max_{i}\max_{j}|s_{j}^{*}-s_{\text{ij}}\right|}$$

where, $s_{j}^{*}$ denotes the optimal value of indicator j, ρ = 0.5 is the distinguishing coefficient and hesitation-sensitivity factor.

##### Final comprehensive evaluation

Finally, combining the DEMATEL-derived indicator weights w_i_, the overall evaluation score of plant i is obtained as

(Equation 22) $$S_{i}=\sum_{j=1}^{n}w_{i}\;\xi_{\text{ij}}$$

where S_i_ represents the comprehensive GPFHFS score of plant.

### Quantification and statistical analysis

Quantitative analyses were conducted after qualitative coding and indicator construction. The unit of analysis varied by stage. For the semi-structured interviews and pairwise influence scoring, *n* = 127, where n represents the number of respondents, including experts, SHP plant managers, and local residents. For the case-plant evaluation, *n* = 5, where n represents the five representative refurbished SHP plants selected from the major regions of China.

Pairwise influence relationships among water-energy-food benefits were quantified using the GW-DEMATEL model. Respondents scored influence strength on a 0–4 scale, where 0 indicated no influence and 4 indicated very strong influence. Individual scoring matrices were aggregated, normalized, and used to calculate total-relation matrices, causal attributes, and integrated weights. The comprehensive benefits of the five representative SHP plants were then evaluated using the GPFHFS method based on stakeholder-group evaluation information and benefit-dimension weights.

Because this study adopted an exploratory multi-stakeholder cognitive evaluation design rather than a randomized experimental or large-sample statistical inference design, no hypothesis-testing-based statistical tests were performed. Exact sample sizes, definitions of n, scoring scales, and calculation procedures are provided in the STAR Methods, Results, figures, and supplemental tables.

All calculations were implemented using Microsoft Excel 2021 and MATLAB R2023b. Spatial visualization was performed using ArcGIS Desktop 10.6.2.

## References

[1] Yüksel I. (2010). Hydropower for sustainable water and energy development. Renew. Sustain. Energy Rev..

[2] Klein S.J.W., Fox E.L.B. (2022). A review of small hydropower performance and cost. Renew. Sustain. Energy Rev..

[3] Luo Q., Xu Y.Y., Zhang W., Wu R., Lin X., Cai Q., Chiu M.C. (2024). Research status and future agenda in small hydropower from the perspective of Sustainable Development Goals. ACS ES&T Water.

[4] Sun X., Wang X., Liu L., Fu R. (2019). Development and present situation of hydropower in China. Water Policy.

[5] Hennig T., Harlan T. (2018). Shades of green energy: Geographies of small hydropower in Yunnan, China and the challenges of over-development. Glob. Environ. Change.

[6] Cui Z., Cai X. (2017). Green small hydropower in China: Practices and drivers. J. Renew. Sustain. Energy.

[7] de Souza E.G., Nadaleti W.C., Thue P.S., dos Santos M.C. (2024). Exploring the capacity and economic viability of green hydrogen production by utilising surplus energy from wind farms and small hydropower plants in Southern Brazil. Int. J. Hydrogen Energy.

[8] Yang Y., Yang B., Xin Z. (2024). Green finance development, environmental attention and investment in hydroelectric power: From the perspective of environmental protection law. Financ. Res. Lett..

[9] Rachmawatie D. (2024). Investigating the smart community empowerment in the utilization of micro hydro power plants (PLTMH): Enhancing the welfare of rural community. E3S Web Conf..

[10] Degani M. (2024). Nun of the river: The material and spiritual economies of small hydropower in rural Tanzania. Crit. Anthropol..

[11] Haulle E., Ndimbo G.K. (2024). Sustainable rural electrification: Small hydropower stations, electrification and rural welfare improvement in Tanzania. Int. J. Dev. Issues.

[12] Lin Z., Qi X., Li M., Duan Y., Gao H., Liu G., Khan S., Mu H., Cai Q., Messyasz B., Wu N. (2024). Differential impacts of small hydropower plants on macroinvertebrate communities upstream and downstream under ecological flow. J. Environ. Manage..

[13] Hedger R.D., Kenawi M.S., Sundt-Hansen L.E., Bakken T.H., Sandercock B.K. (2025). Evaluating environmental impacts of micro, mini and small hydropower plants in Norway. J. Environ. Manage..

[14] Albrecht E., Isaac R., Räsänen A. (2024). Legal and political arguments on aquatic ecosystem services and hydropower development: A case study on Kemi River basin, Finland. Ecosyst. Serv..

[15] Moschidis O., Arabatzis G. (2013). SHP stations and integrated rural development: A multivariate statistical approach. Int. J. Green Econ..

[16] Butchers J., Williamson S., Booker J. (2021). Micro-hydropower in Nepal: Analysing the project process to understand drivers that strengthen and weaken sustainability. Sustainability.

[17] Coban H.H., Sauhats A. (2022). Optimization tool for small hydropower plant resource planning and development: A case study. J. Adv. Res. Nat. Appl. Sci..

[18] Melina A.B., Nzanywayingoma F., Kabiri C., Rushingabigwi G. (2024). 2024 International Conference on Green Energy, Computing and Sustainable Technology (GECOST).

[19] Li J., Tian G., Wu Z., Jin Y., Zhou T. (2025). Unveiling benefits: A framework for analyzing small hydropower refurbishment activities. Renew. Sustain. Energy Rev..

[20] Tidjani H., Kenfack J., Hamandjoda O., Wankie M., Raïssa M., Calvin N. (2025). Fuzzy MCDM: An approach for selecting micro turbine technology in a micro hydropower project in Cameroon. Int. J. Sustain. Green Energy.

[21] Kaldellis J.K., Vlachou D.S., Korbakis G. (2005). Techno-economic evaluation of small hydro power plants in Greece: A complete sensitivity analysis. Energy Policy.

[22] Pang M., Zhang L., Wang C., Liu G. (2015). Environmental life cycle assessment of a small hydropower plant in China. Int. J. Life Cycle Assess..

[23] Zhang L.X., Pang M.Y., Wang C.B. (2014). Emergy analysis of a small hydropower plant in southwestern China. Ecol. Indic..

[24] Boavida I., Costa M.J., Santos J.M. (2025). Community perceptions and ecosystem services provided by small hydropower plants. Environ. Dev..

[25] Gioia D.A., Corley K.G., Hamilton A.L. (2013). Seeking qualitative rigor in inductive research: Notes on the Gioia methodology. Organ. Res. Methods.

[26] Tang X., Wei G. (2019). Multiple attribute decision-making with dual hesitant Pythagorean fuzzy information. Cogn. Comput..

[27] Hoff H. (2011). Background Paper for the Bonn 2011 Conference: The Water, Energy and Food Security Nexus.

[28] Zhang X., Li H.Y., Deng Z.D., Ringler C., Gao Y., Hejazi M.I., Leung L.R. (2018). Impacts of climate change, policy and Water-Energy-Food nexus on hydropower development. Renew. Energy.

[29] McNabola A., Mérida García A., Rodríguez Díaz J.A. (2022). The role of micro-hydropower energy recovery in the Water-Energy-Food nexus. Environ. Sci. Proc..

[30] Allouche J., Middleton C., Gyawali D. (2019).

[31] Naidoo D., Nhamo L., Mpandeli S., Sobratee N., Senzanje A., Liphadzi S., Slotow R., Jacobson M., Modi A.T., Mabhaudhi T. (2021). Operationalising the water-energy-food nexus through the theory of change. Renew. Sustain. Energy Rev..

[32] Jones-Crank J.L. (2025). A typology of water-energy-food nexus research. Environ. Sci. Policy.

[33] Afshar A., Soleimanian E., Akbari Variani H., Vahabzadeh M., Molajou A. (2022). The conceptual framework to determine interrelations and interactions for holistic Water, Energy, and Food Nexus. Environ. Dev. Sustain..

[34] Vahabzadeh M., Afshar A., Molajou A. (2023). Energy simulation modeling for water-energy-food nexus system: A systematic review. Environ. Sci. Pollut. Res. Int..

[35] Vahabzadeh M., Afshar A., Molajou A. (2023). Framing a novel holistic energy subsystem structure for water-energy-food nexus based on existing literature (basic concepts). Sci. Rep..

[36] Do P., Tian F., Zhu T., Zohidov B., Ni G., Lu H., Liu H. (2020). Exploring synergies in the water-food-energy nexus by using an integrated hydro-economic optimization model for the Lancang-Mekong River basin. Sci. Total Environ..

[37] Gioia D. (2021). A systematic methodology for doing qualitative research. J. Appl. Behav. Sci..

[38] Si S.L., You X.Y., Liu H.C., Zhang P. (2018). DEMATEL technique: A systematic review of the state-of-the-art literature on methodologies and applications. Math. Probl Eng..

[39] Khan M.S.A., Abdullah S., Ali A., Siddiqui N., Amin F. (2017). Pythagorean hesitant fuzzy sets and their application to group decision making with incomplete weight information. J. Intell. Fuzzy Syst..

[40] Mahmoudi A., Mi X., Liao H., Feylizadeh M.R., Turskis Z. (2020). Grey best-worst method for multiple experts multiple criteria decision making under uncertainty. Informatica.

