# Supplementary material for: An integrated assessment of water-energy-food benefits from small hydropower refurbishment in rural China
Source: iScience. 2026 May 28;29(6):116133. doi: 10.1016/j.isci.2026.116133 (PMC13233801; doi:10.1016/j.isci.2026.116133)
Supplement: Document S1. Figure S1 and Table S1 [file mmc1.pdf]

iScience, Volume *29*

Supplemental information

An integrated assessment of water-energy-food benefits from small hydropower refurbishment in rural China

Jiawen Li, Guiliang Tian, Zheng Wu, Xinyu Liu, Qing Xia, and Canran Lu

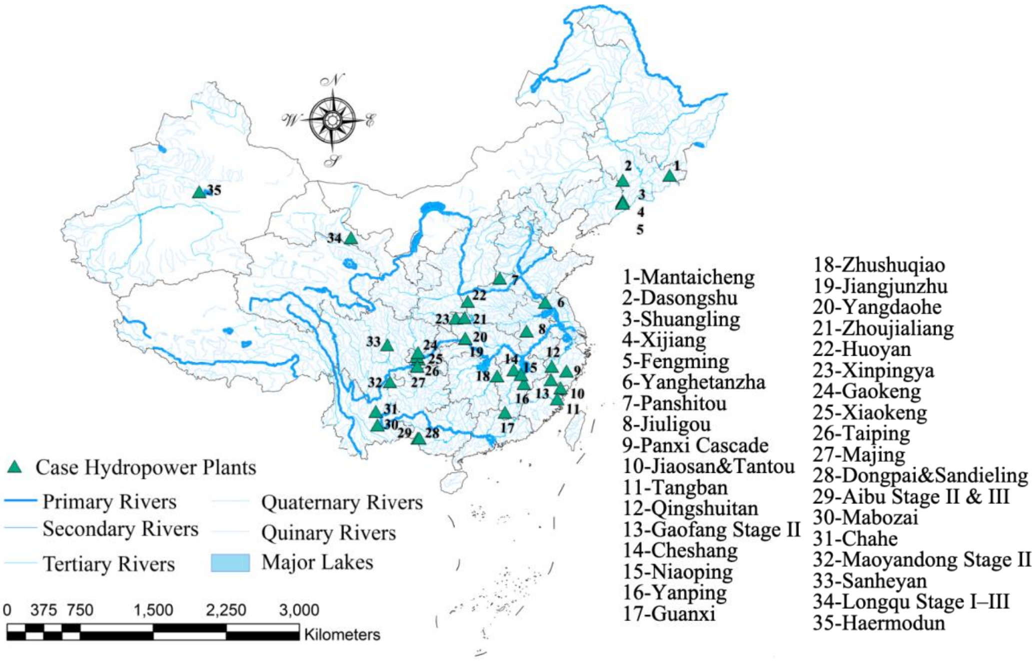

Figure S1 Distribution of the 35 case SHP plants

Table S1 Basic characteristics of the interview sample

Indicate	Options	Frequency	Percentage
Gender	Male	82	64.57%
	Female	45	35.43%
Age	22–30 years old	23	18.11%
	31–40 years old	41	32.28%
	Over 40 years old	63	49.61%
Work seniority	1-2 years	21	16.54%
	2-3 years	44	34.65%
	More than 3 years	62	48.82%
Categories	SHP managers	41	32.28%
	SHP experts	7	5.51%
	Nearby risidents	79	62.20%
